# Liraglutide Modifies Gut Microbiota Without Modulating Doxorubicin-Induced Toxicity in Rats

**DOI:** 10.3390/antiox15050538

**Published:** 2026-04-24

**Authors:** Carolina R. Tonon, Marina G. Monte, Paola S. Ballin, Anderson S. S. Fujimori, Natália F. Ferreira, Nayane M. Vieira, Lara P. Carreira, Maria A. M. Rodrigues, Josias Rodrigues, Luiz Almeida Junior, Luiz C. Di Stasi, Andrey Santos, Daniela O. Magro, Marcos F. Minicucci, Leonardo A. M. Zornoff, Marina P. Okoshi, Sergio A. R. Paiva, Bertha F. Polegato

**Affiliations:** 1 Internal Medicine Department, Botucatu Medical School, São Paulo State University (UNESP), Botucatu 18618-687, Brazil; marina.monte@unesp.br (M.G.M.); paola.balin@unesp.br (P.S.B.); seiji.fujimori@unesp.br (A.S.S.F.); natalia.fernanda@unesp.br (N.F.F.); n.vieira@unesp.br (N.M.V.); lara.pompiani@unesp.br (L.P.C.); marcos.minicucci@unesp.br (M.F.M.); leonardo.zornoff@unesp.br (L.A.M.Z.); marina.okoshi@unesp.br (M.P.O.); sergio.paiva@unesp.br (S.A.R.P.); bertha.polegato@unesp.br (B.F.P.); 2 Pathology Department, Botucatu Medical School, São Paulo State University (UNESP), Botucatu 18618-687, Brazil; maria.marchesan@unesp.br; 3 Laboratory of Medical Bacteriology, Department of Microbiology and Immunology, Institute of Biosciences, São Paulo State University (UNESP), Botucatu 18618-689, Brazil; josias.rodrigues@unesp.br; 4 Laboratory of Phytomedicines, Pharmacology and Biotechnology (PhytoPharmaTech), Department of Biophysics and Pharmacology, Institute of Biosciences, São Paulo State University (UNESP), Botucatu 18618-689, Brazil; luiz.domingues@unesp.br (L.A.J.); luiz.stasi@unesp.br (L.C.D.S.); 5 Faculty of Medical Science, State University of Campinas (UNICAMP), Campinas 13083-894, Brazil; andreysts@unicamp.br (A.S.); dmagro@unicamp.br (D.O.M.)

**Keywords:** doxorubicin, intestinal toxicity, gut microbiota, short-chain fatty acids, GLP-1

## Abstract

Doxorubicin is an effective chemotherapeutic agent, but it causes gastrointestinal toxicity that impairs treatment efficacy and quality of life. This study investigated the effects of liraglutide, a GLP-1 analog, on acute doxorubicin-induced gut toxicity in rats. Sixty male Wistar rats were assigned to four groups: Control (C), Doxorubicin (D), Liraglutide (L), and Doxorubicin + Liraglutide (DL). Groups L and DL received liraglutide (0.6 mg/kg, s.c.) for two weeks. D and DL were given a single dose of doxorubicin (20 mg/kg, i.p). After 48 h, the distal colon, feces, and blood were collected. Results: Doxorubicin caused crypt disruption, goblet cell loss, apoptosis, and reduced fecal short-chain fatty acids. Levels of TNF-α, NF-κB, Bcl-2, TLR4, and antioxidant enzymes were unchanged among groups. Microbiota analysis showed similar α-diversity but altered β-diversity. Doxorubicin reduced Bacteroidetes and increased Proteobacteria, with higher Arcanobacterium and Clavibacter genera abundance. Liraglutide alone decreased Bacteroidetes and increased Corynebacterium and Actinobaculum genera. Combined treatment showed no significant effects. We conclude that acute doxorubicin administration induces intestinal structural damage, reduces short-chain fatty acids, and changes microbiota composition. Although liraglutide alters microbial profiles, it does not attenuate doxorubicin-induced gut toxicity.

## 1. Introduction

Cancer is a highly prevalent disease and a leading cause of death worldwide, accounting for almost ten million deaths in 2020. Doxorubicin is a very potent chemotherapy agent used for more than forty years in the treatment of several types of solid and hematological cancers [1].

However, in addition to its anti-tumoral effect on neoplastic cells, it may also affect healthy cells in various organs and tissues, such as the heart, kidneys, liver, bone marrow, and gastrointestinal tract, limiting its use [2].

The gastrointestinal (GI) tract is particularly vulnerable to the cytotoxic effects of doxorubicin due to its rapid cellular turnover. It is estimated that around 40% of individuals undergoing chemotherapy experience GI-related adverse effects [3]. Among the most frequently reported complications are nausea, vomiting, abdominal discomfort, and diarrhea, all of which can significantly impair nutritional intake and quality of life [4]. These gastrointestinal disturbances may contribute to extended hospital stays, increased need for parenteral nutrition, and a higher risk of infections, ultimately exacerbating patient morbidity and mortality [5,6].

The pathophysiology of chemotherapy-induced intestinal toxicity is multifactorial and involves both direct epithelial injury and indirect alterations in gut microbiota composition and function [7]. The gut microbiota consists of a highly dynamic population of microorganisms that plays a crucial role in maintaining intestinal barrier integrity and metabolic homeostasis. These microorganisms contribute to host health through multiple mechanisms, including vitamin synthesis, competitive exclusion of pathogenic bacteria, modulation of immune responses, regulation of mucin production, and reinforcement of epithelial junctional proteins such as occludins, claudins, junctional adhesion molecules (JAM), and zonula occludens (ZO-1). In addition, the gut microbiota is essential for the digestion of complex macromolecules and for the production of short-chain fatty acids (SCFAs), including acetate, propionate, and butyrate, which serve as key energy substrates for colonocytes and play a fundamental role in maintaining mucosal integrity [8,9].

Disruption of this microbial ecosystem, commonly referred to as dysbiosis, has been associated with increased production of pro-inflammatory cytokines, reduced SCFA synthesis, and impairment of intestinal barrier function [10,11]. Under physiological conditions, the intestinal epithelium acts as a selective barrier that prevents the translocation of bacteria and endotoxins into the systemic circulation. However, when epithelial integrity is compromised, luminal microorganisms and microbial-derived products may reach the bloodstream, triggering systemic inflammatory responses. This process has been implicated not only in intestinal pathology but also in the exacerbation of extra-intestinal complications, including doxorubicin-induced cardiotoxicity [12,13,14].

Emerging evidence further indicates that gut microbiota alterations are closely linked to the pathogenesis of several cardiovascular and metabolic disorders, such as hypertension, obesity, dyslipidemia, insulin resistance, atherosclerosis, and heart failure. Consequently, strategies aimed at preserving intestinal barrier function and maintaining microbiota homeostasis have gained increasing attention as potential approaches to attenuate systemic inflammation and reduce treatment-related complications [13,14,15].

Liraglutide, a glucagon-like peptide-1 (GLP-1) receptor agonist, is widely used in the management of type 2 diabetes mellitus and obesity [16]. Beyond its metabolic actions, growing experimental evidence indicates that GLP-1 analogs may exert protective effects on the gastrointestinal tract. These agents have been associated with the preservation of intestinal epithelial integrity through multiple complementary mechanisms, including the promotion of deeper intestinal crypt architecture, enhancement of epithelial cell turnover, and strengthening of intercellular junctional complexes, thereby contributing to reduced intestinal permeability. In addition, GLP-1 receptor activation has been linked to increased mucus secretion and improved barrier function, which may help maintain mucosal homeostasis under conditions of metabolic or inflammatory stress.

Furthermore, recent studies suggest that GLP-1 analogs may influence the composition and metabolic activity of the gut microbiota, potentially modulating host–microbe interactions that are relevant to intestinal physiology and systemic metabolic regulation. Collectively, these findings support the concept that GLP-1–based therapies may have broader biological effects extending beyond glucose metabolism, including a potential role in protecting intestinal structure and function [17,18,19,20,21,22,23,24].

Numerous studies have investigated the impact of various agents on the intestinal microbiota with the goal of mitigating or preventing toxicity induced by doxorubicin [25,26,27,28,29,30,31,32,33,34]. Nevertheless, to date, no research has specifically explored the role of GLP-1 analogs in modulating the gut microbiota to counteract or reduce doxorubicin-associated intestinal damage.

In this study, we proposed that liraglutide attenuates doxorubicin-induced intestinal toxicity and restores the composition and function of the gut microbiota in rats. Therefore, the aim of our study was to evaluate the effects of liraglutide on intestinal histology, oxidative stress, inflammation, and apoptosis, as well as on gut microbiota function and composition in rats with acute doxorubicin-induced intestinal toxicity. Detailed results for myocardial morphometric evaluation, oxidative stress analyzes, and protein expression have been previously published [35,36,37].

## 2. Materials and Methods

### 2.1. Study Design

**Figure 1:**
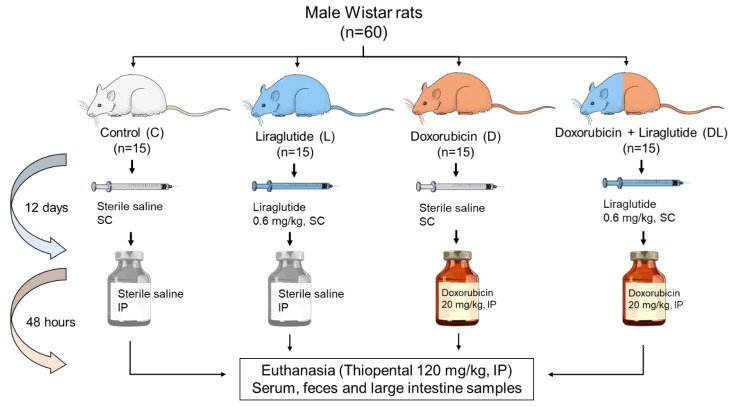
Experimental design. C: control; D: doxorubicin; L: liraglutide; DL: doxorubicin + liraglutide; SC: subcutaneous; IP: intraperitoneal. Created by the authors (Power Point, Microsoft).

Sixty male Wistar rats, each weighing between 250 and 300 g, were randomly divided into four groups of 15 animals: control (C), doxorubicin (D), liraglutide (L), and a combination of doxorubicin and liraglutide (DL). All animals were housed under controlled conditions with a 12-h light/dark cycle at a temperature of 23 ± 2 °C, with free access to standard chow and water. Groups L and DL received liraglutide (Novo Nordisk, Brazil) at a dose of 0.6 mg/kg subcutaneously once daily for 14 days. In contrast, groups C and D received equivalent volumes of saline. On the 12th day, groups D and DL were given a single intraperitoneal injection of doxorubicin (Eurofarma, São Paulo, Brazil) at 20 mg/kg, while groups C and L received an intraperitoneal injection of saline in the same volume (Figure 1).

Fecal samples from each animal were collected the day before the start of the experiment and at the end of the experimental period. The rats were individually placed on a sanitized bench with 70% ethanol until evacuation, and the feces samples were immediately collected and stored at −80 °C.

At the conclusion of the experiment, the animals were euthanized using thiopental administered intraperitoneally at a dose of 120 mg/kg. Blood samples were obtained via decapitation and subsequently centrifuged at 1300× *g* for 10 min at 21 °C. The resulting serum was collected and stored at −80 °C for later analysis. Sections of the large intestine were excised and rinsed in cold saline solution. One portion of the tissue was processed for histological examination, while the other was frozen in liquid nitrogen and preserved at −80 °C.

### 2.2. Microbiota Analysis

Fecal microbial profiling was conducted through sequence comparison of the V6 region of the 16S rDNA gene (16SV6rDNA) from each sample against reference sequences available in public databases.

#### 2.2.1. Polymerase Chain Reaction (PCR) and Amplicon Sequencing

Primers were specifically designed to include adapter sequences; the reverse primers also incorporated unique barcodes for sample identification. The resulting amplicons were assessed via electrophoresis on a 1% agarose gel (Sigma St. Louis, MO, USA) prepared with Tris-acetate-EDTA buffer. A DNA ladder was used as a molecular weight reference to confirm successful and specific amplification.

Purification of the amplicons was carried out using the AMPure XP magnetic bead system (Beckman Coulter, Brea, CA, USA), followed by DNA quantification with a Qubit 3.0 Fluorometer (Invitrogen, Waltham, MA, USA) and the Qubit dsDNA HS Assay Kit (Invitrogen). A pooled amplicon library was then constructed at a final concentration of 26 pM, ensuring equimolar representation of each sample.

This library underwent emulsion PCR, in which primers and other PCR reagents were combined with Ion Sphere Particles (ISPs) and oil, then amplified in an Ion OneTouch Thermocycler (ThermoFisher, Waltham, MA, USA). Unsuccessful ISPs and residual reagents were removed, enriching the preparation with ISPs containing amplified DNA suitable for sequencing.

Following enrichment, sequencing primers were annealed to the DNA, and the mixture was combined with Taq DNA polymerase. The final preparation was loaded onto a 314 v2-BC chip, which was inserted into an Ion Personal Genome Machine (PGM) sequencer. The sequencer was preconditioned, with pH balance and incorporation of necessary reagents (dNTPs). Sequencing output was automatically stored as *.fastq files on a dedicated server.

#### 2.2.2. Processing and Analysis of Sequences

Raw sequencing data were processed to remove low-quality reads, chimeric sequences, and residual primer, barcode, and adapter fragments. Quality metrics were reported by the server, including QC20 scores, DNA fragmentation patterns, sequence length distribution, read counts, chip loading efficiency, and ISP enrichment levels. A separate *.fastq file was generated for each individual sample.

Data analysis proceeded in two stages. The first involved sequence organization and operational taxonomic unit (OTU) assignment. The sequencing depth was 1.5 ± 0.9 million bases and we used relative abundance for normalization. Sequences were demultiplexed and analyzed on the Qiita platform, then compared against the Silva 119 database (Bremen, Germany) for taxonomic identification. In the second stage, the OTU data were compiled into a *.csv file. Due to formatting errors, the file initially contained inconsistencies; after correction, the dataset was subjected to statistical analysis. The data for this study have been deposited in the European Nucleotide Archive (ENA) at EMBL-EBI under the accession number PRJEB90078 (https://www.ebi.ac.uk/ena/browser/view/PRJEB90078, accessed on 5 June 2025).

### 2.3. Short-Chain Fatty Acids (SCFAs)

After thawing, 100 mg of feces was suspended in 1 mL of 0.5% phosphoric acid, homogenized using a vortex for approximately 1 min, and then centrifuged for 10 min at 17,950× *g*. Each mL of the supernatant was combined with 1 mL of ethyl acetate for 1 min and centrifuged for 10 min at 17,950× *g*. Prior to the analysis, 1 mL of the organic phase was transferred into a tube, and octanoic acid was added as an internal standard (IS) at a final concentration of 755 µg/mL. The IS was used to correct for injection variability between samples and minor changes in instrument response. Three independent replicate extractions were performed for each sample, with two injections per extraction.

Chromatographic analysis was conducted using a FOCUS Thermo Scientific Gas Chromatography Mass Spectrometry, equipped with an automatic liquid sampler (Thermo-Triplus DUO), and coupled to a Thermo ISQ 230ST mass detector (ThermoFisher, MA, USA). The Gas Chromatograph (GC) was fitted with a high-polarity polyethylene glycol fused silica capillary column (TG-WAXMS, 30 m, 0.25 mm id, 0.25 μm film thickness), with helium as the carrier gas. Here, the injection was done in split mode with an injection volume of 1 µL, a split flow of 300 mL/min, and a temperature of 250 °C. A glass liner with a glass wool plug at the lower end was used to prevent contamination of the GC column with non-volatile fecal material. After every ten fecal samples injected, a blank sample containing ethyl acetate was inserted to check for memory effects. The column temperature was initially set to 80 °C, then increased to 120 °C at 50 °C/min, to 160 °C at 70 °C/min, and held at that temperature for 50 s. It was then increased to 180 °C at 70 °C/min and held for 1 min, and finally to 210 °C at 70 °C/min and held for 1 min. The solvent delay was set to 3.5 min, and the detector was operated in electron impact ionization mode (electron energy 70 eV), scanning in the 30–250 *m*/*z* range. SCFA identification was based on the retention times of standard compounds and the assistance of the National Institute of Standards & Technology 08 and online libraries. The peaks were quantified as the relative abundance of the total ionic count using the internal standard. The concentration (mg/mL) of each SCFA was calculated using the linear regression equations (R^2^ ≥ 0.99) from the corresponding standard curves obtained from 12 different concentrations.

### 2.4. Lipopolysaccharides

The serum concentration of lipopolysaccharides (LPS) was determined by enzyme-linked immunossorbent assays (ELISA) using the commercial kit from ELK Biotechnology (Sugar Land, TX,, USA). LPS are components of the bacterial cell membrane and serve as damage markers to intestinal barrier integrity. Serum protein concentrations were measured using the Bradford method (Biorad, Hercules, CA, USA). Briefly, 96-well plates were coated with a solution containing purified capture antibodies, diluted in phosphate-buffered saline (PBS) and incubated at room temperature overnight. After successive washes with PBS-Tween 20 (0.05%), 300 μL of blocking solution, consisting of PBS containing 1% albumin, was added, and the plates were incubated for 2 h at room temperature. The plates were then washed again and incubated for a further 2 h at room temperature with the samples and respective LPS standard curves diluted 2-fold in PBS buffer containing 1% albumin. After this incubation, the plates were washed and incubated with biotinylated rat antibodies for another 2 h at room temperature. Afterward, the plates were incubated with streptavidin, diluted 1:200 in PBS buffer containing 1% albumin, for 20 min at room temperature. Finally, the plates were washed and developed with o-phenylenediamine dihydrochloride (Sigma-Aldrich, St. Louis, MO, USA). The reaction was stopped by adding 16% sulfuric acid, and the reading was performed at 450 and 490 nm.

### 2.5. Histology

Segments of the large intestine were fixed in 10% buffered formalin and subsequently embedded in paraffin. Tissue sections of 5 µm thickness were obtained and stained with hematoxylin and eosin (H&E) for histological evaluation. Morphological assessments were carried out using a light microscope at 40× magnification, equipped with a video camera connected to a computer for image capture and analysis. The histological evaluation was performed by an experienced pathologist that did not known the groups.

### 2.6. Oxidative Stress

Oxidative stress in intestinal tissue was evaluated by quantifying malondialdehyde (MDA), a marker of lipid peroxidation, and protein carbonyl content, indicative of protein oxidation.

MDA levels were determined using the thiobarbituric acid reactive substances (TBARS) assay. In brief, 200 µL of tissue homogenate supernatant was mixed with 500 µL of a reagent containing 0.67% thiobarbituric acid, 10% trichloroacetic acid, and 0.25 M hydrochloric acid. The mixture was centrifuged at 13,285× *g* for 10 min, after which the supernatant was incubated in a water bath at 100 °C for 45 min. Following incubation, 200 µL of the reaction mixture was transferred to a microplate, and absorbance was measured at 532 nm and 600 nm using a Spectra Max 190 microplate reader (Molecular Devices, San José, CA, USA).

Protein carbonylation was measured by reacting 100 µL of tissue supernatant with 100 µL of 2,4-dinitrophenylhydrazine (DNPH). After a 10-min incubation at room temperature, 50 µL of 6 M sodium hydroxide (NaOH) was added, followed by another 10-min incubation. Absorbance was recorded at 450 nm using the same microplate reader. Protein oxidation levels were calculated using a molar extinction coefficient of 22,000 M^−1^·cm^−1^.

### 2.7. Antioxidant Enzymes

The technique for assessing superoxide dismutase (SOD) activity was based on the inhibition of the reaction between the superoxide anion radical and pyrogallol. Samples of 100 mg of intestinal tissue were homogenized in a 0.01 M sodium phosphate buffer at a pH of 7.4 and centrifuged for 30 min at −4 °C. Afterward, a buffer solution (Tris base 50 mmol/L; EDTA 1 mmol/L at pH 8.5) and pyrogallol 2.6 mmol/L (in hydrochloric acid at 10 mmol/L) were added. Oxidation of pyrogallol leads to the formation of a colored product, detected by spectrophotometry at 420 nm allowing the evaluation of SOD activity. The results were expressed in U SOD/mg of protein.

Catalase activity was measured by evaluating hydrogen peroxide consumption. The decrease in absorbance was assessed at a wavelength of 240 nm, where hydrogen peroxide exhibits the highest absorption. The following reagents were used: sodium phosphate buffer 50 mmol/L + EDTA 1 mmol/L (pH 7.0) and hydrogen peroxide 0.3 mol/L. Concentration was expressed in mol/mg of protein.

### 2.8. Western Blot

Samples of large intestine tissue (100 mg) were homogenized using glass beads in an extraction buffer containing 100 mM NaCl, 1% Triton X-100, 0.5% sodium deoxycholate, 0.1% SDS, 10% glycerol, 10 mM Tris (pH 7.4), 1 mM EDTA, 1 mM sodium orthovanadate, 10 mM NaF, and a protease inhibitor cocktail (P2714, Sigma-Aldrich, MO, USA). Homogenization was performed using a Bullet Blender (Next Advance, Troy, NY, USA). The homogenate was centrifuged at 150,000× *g* for 20 min at 4 °C, and the supernatant was collected for analysis. Total protein concentration was determined using the Bradford assay. Protein samples were mixed with Laemmli buffer (Sigma-Aldrich) and subjected to SDS-PAGE using a Mini-Protean 3 Electrophoresis Cell (Bio-Rad, Hercules, CA, USA). Stacking and resolving gels were prepared with Tris-HCl buffer (240 mM, pH 6.7 and 8.9, respectively), polyacrylamide, glycerol, APS, and TEMED. Electrophoresis was carried out initially at 50 V for 30 min, followed by 120 V for 2.5 h at 4 °C using a running buffer composed of 0.25 M Tris, 192 mM glycine, and 1% SDS. Proteins were transferred onto nitrocellulose membranes using the Mini-TransBlot system (Bio-Rad) in a transfer buffer containing 25 mM Tris, 192 mM glycine, 20% methanol, and 1% SDS. To block nonspecific binding, membranes were incubated in a 5% skim milk solution prepared in a basal buffer (1 M Tris, 5 M NaCl, and Tween 20, pH 8.0). Primary antibodies against tumor necrosis factor-α (TNF-α), total and phosphorylated nuclear factor kappa B (NF-κB), toll-like receptor 4 (TLR-4), and B-cell lymphoma 2 (BCL-2) (Santa Cruz Biotechnology, Dallas, TX, USA) were diluted in the same basal solution and incubated with the membranes overnight at 4 °C. After washing three times, membranes were incubated for 1.5 h at room temperature with a rabbit anti-mouse IgG secondary antibody (ab6728, Abcam, Cambridge, UK ), followed by additional washes. Detection was performed via chemiluminescence using the ImageQuant LAS imaging system (General Electric, Boston, MA, USA). Densitometric analysis was carried out using Gel-Pro 32 software (Media Cybernetics, Rockville, MD, USA). Regarding normalization, the constitutive proteins GAPDH and β-tubulin were altered in this experimental model; therefore, we could not use them. Thus, the normalization of the proteins was performed by dividing the protein of interest by the total protein content obtained through Ponceau staining, as previously described in the literature.

### 2.9. Statistical Analysis

Data are expressed as means ± standard deviation. Group comparisons were conducted using a generalized linear model (GLM) with a gamma distribution, considering the treatment groups as independent variables. This model allows the evaluation of the isolated effect of each treatment, regardless of the influence of the other factor, as well as the assessment of potential interaction effects between treatments. Three p-values were reported: pDxL for the interaction between doxorubicin and liraglutide, pD for the main effect of doxorubicin, and pL for the main effect of liraglutide. When a significant interaction between factors was detected (D × L < 0.05), post hoc multiple comparisons were performed to identify specific group differences. In the absence of a significant interaction (D × L > 0.05), marginal results were analyzed to determine the independent effects of doxorubicin and liraglutide. For microbiota α-diversity, we used the paired t-test, while for β-diversity, we performed the principal coordinate analysis (PCoA) at the species level with Bray–Curtis’s distance followed by PERMANOVA. Afterward, LDA (linear discriminant analysis) was performed to identify bacterial species contributing to alterations in gut microbiota composition. We used the free access Jamovi software, Version 2.3, at https://www.jamovi.org for GLM and t-test and the QIITA platform (San Diego, CA, USA), which relies on QIIME 2 workflows, for microbiota analyses.

## 3. Results

### 3.1. Body Weight and Food Ingestion

As previously reported in our first paper on this topic [35], initial body weight did not differ between groups (C 287 ± 22; D 288 ± 29; L 289 ± 31; DL 290 ± 31 g; pD = 0.86; pL = 0.77; pDxL = 0.98). However, the final body weights were smaller in rats treated with doxorubicin (D and DL) and treated with liraglutide (L and DL) (C 332 ± 26; D 300 ± 34; L 303 ± 25; DL 268 ± 28; pD < 0.001; pL < 0.001; pDxL = 0.87). After doxorubicin administration, the rats had a significantly lower food intake than those that did not receive the drug [35]..

### 3.2. Histology

**Figure 2:**
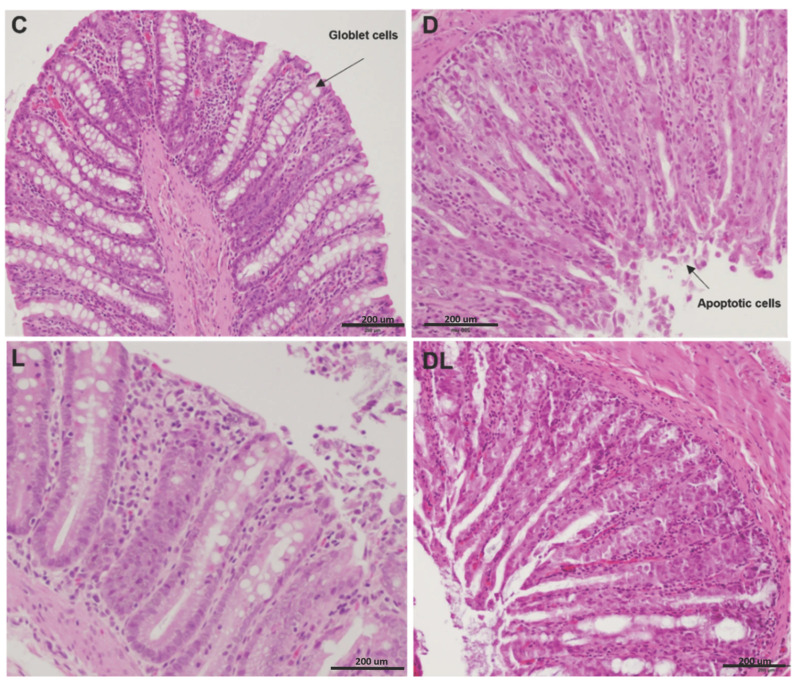
Hematoxylin and eosin-stained histological sections of the large intestine. Optical microscopy with 40× magnification. C: control; D: doxorubicin; L: liraglutide; DL: doxorubicin + liraglutide.

Qualitative histological evaluation of the colon performed by a pathologist using hematoxylin and eosin staining revealed doxorubicin-associated epithelial injury in the D and DL groups (Figure 2). These animals exhibited altered crypt architecture, characterized by crypt shortening and focal crypt loss, along with a reduced number of goblet cells and an increased presence of epithelial cells with apoptotic morphology, including cell shrinkage, hypereosinophilic cytoplasm, and pyknotic or fragmented nuclei. In contrast, colonic sections from the C and L groups showed preserved crypt organization and epithelial morphology, with abundant goblet cells and no evident increase in apoptotic figures. Liraglutide treatment alone did not result in detectable histological alterations compared with controls.

### 3.3. Serum LPS

Serum LPS levels did not differ between groups (C 0.43 ± 0.21; D 0.37 ± 0.16; L 0.27 ± 0.16; DL 0.30 ± 0.25 ng/mL; pD = 0.860; pL = 0.150; pDxL = 0.620).

### 3.4. Western Blot

**Figure 3:**
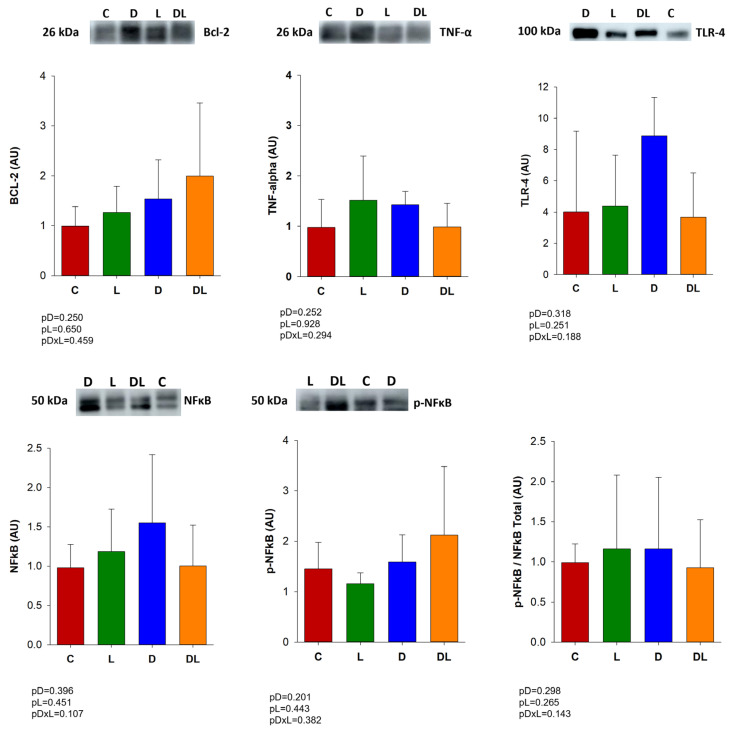
Protein expression by Western blot in the large intestine. C: control (n = 6); D: doxorubicin (n = 6); L: liraglutide (n = 7); DL: doxorubicin + liraglutide (n = 7). TNF-α: tumor necrosis factor α; Bcl-2: B-cell lymphoma 2; NFκB: nuclear factor kappa B; p-NFκB: phosphorylated nuclear factor kappa B; TLR-4: Toll-like receptor 4. Values are expressed as mean ± standard deviation; GLM. pDxL: *p*-value for doxorubicin vs liraglutide interaction; pD: *p*-value for doxorubicin factor; pL: *p*-value for liraglutide factor.

We evaluated proteins related to inflammation and apoptosis by Western blot. Expression of the inflammation-related proteins TNF-α, NFκB and TLR4, and BCL-2 (an anti-apoptotic protein) did not differ between groups (Figure 3).

### 3.5. Oxidative Stress and Antioxidant Enzyme Activities

**Figure 4:**
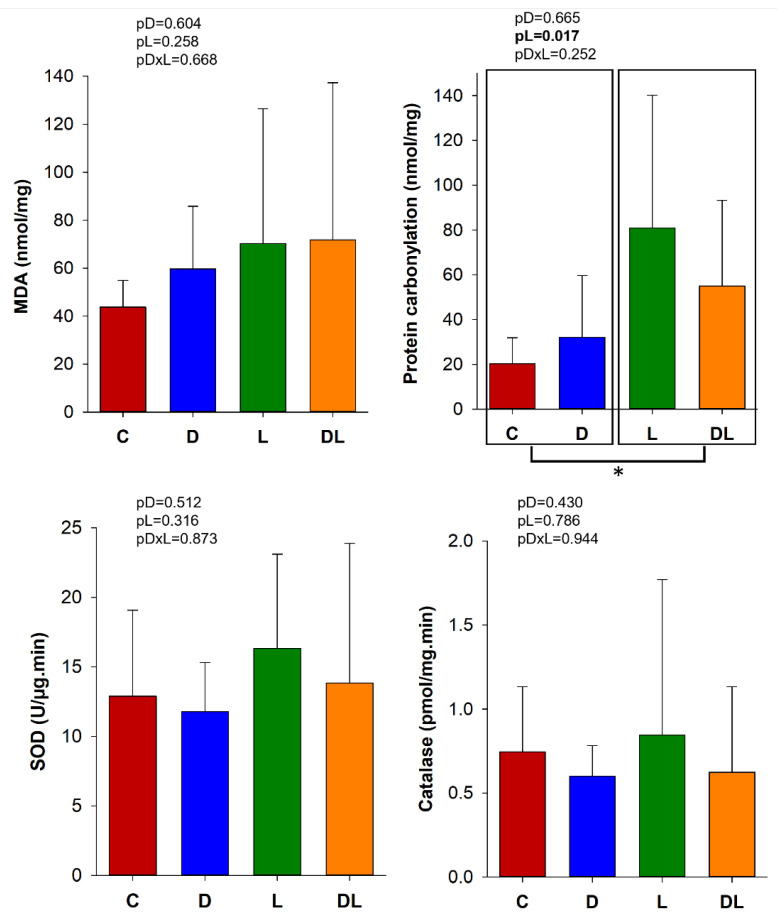
C: control (n = 6); L: liraglutide (n = 7); D: doxorubicin (n = 6); DL: doxorubicin + liraglutide (n = 6). MDA: malondialdehyde, SOD: superoxide dismutase. Data are expressed as means ± SD; GLM; pD: *p*-value for doxorubicin effect; pL: *p*-value for liraglutide effect; pDxL, *p*-value for the interaction between doxorubicin and liraglutide; * different for the liraglutide factor.

MDA concentration reflects lipid peroxidation of cell membranes, and carbonylation protein damage. Protein carbonylation in the large intestine was higher in the rats treated with liraglutide than those that did not receive liraglutide. In addition, MDA concentration and SOD and catalase enzyme activities did not differ between the groups (Figure 4).

### 3.6. Gut Microbiota

**Figure 5:**
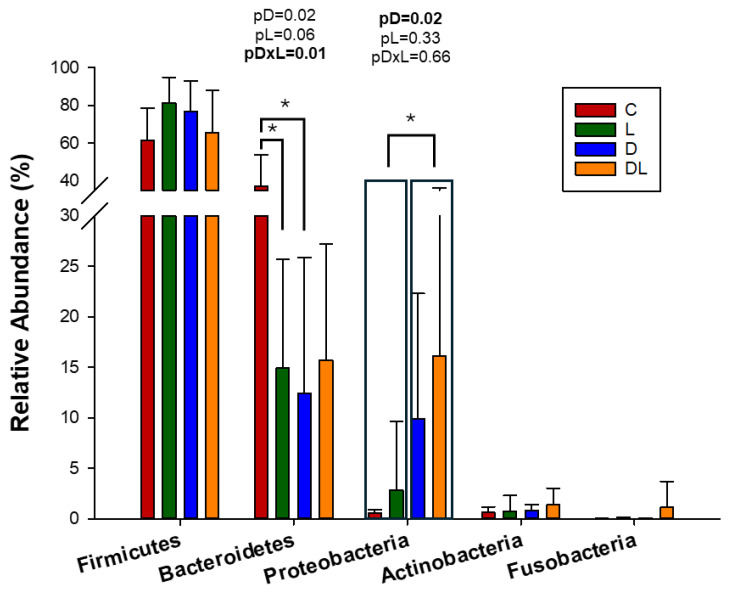
Relative abundance of the gut microbiota at the phyla level after treatment. C: control; D: doxorubicin; L: liraglutide; DL: doxorubicin + liraglutide. Data are expressed as means ± standard deviation; GLM; *p* < 0.05: * Different from comparing group.

A total of 1090 OTUs were obtained from the 60 samples. At the beginning of the experiment, the composition of the gut microbiota was similar between the groups (Figure S1). At the end of the experiment, the relative abundance of Bacteroidetes was lower in groups D and L compared to C, but Proteobacteria was more abundant in the animals treated with doxorubicin (Figure 5).

**Figure 6:**
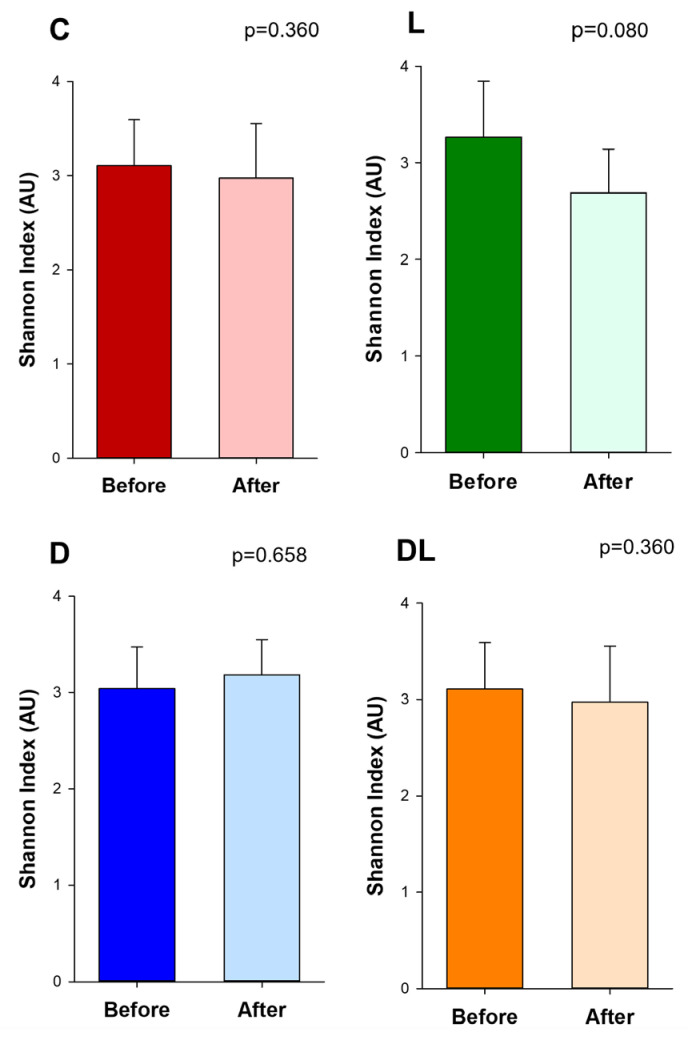
α-diversity. C: control (n = 8); D: doxorubicin (n = 8); L: liraglutide (n = 8); DL: doxorubicin + liraglutide (n = 8). Data are expressed as means ± SD. Paired t test; *p* < 0.05: before vs. after treatment.

**Figure 7:**
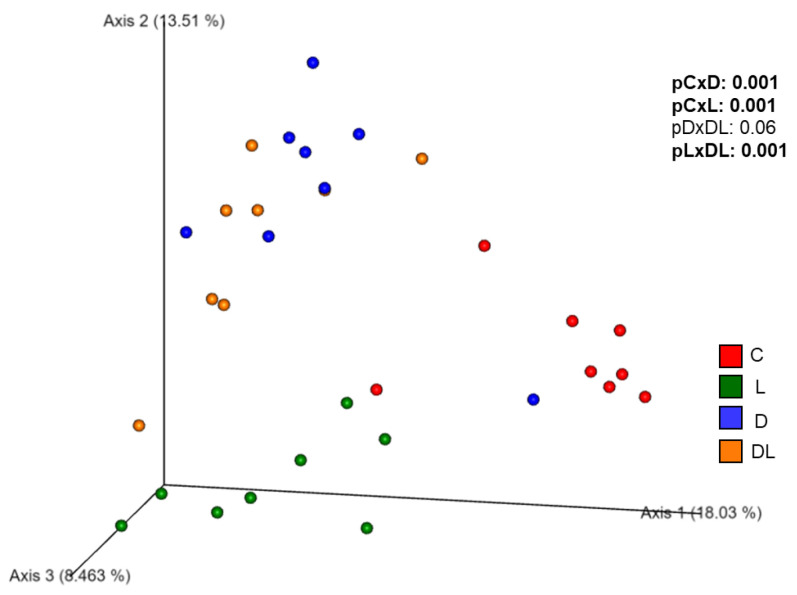
β-diversity after treatment using Principal Coordinate Analysis. C: control; D: doxorubicin; L: liraglutide; DL: doxorubicin + liraglutide. Sample size: 8 per group. *p*-value: PERMANOVA. pCxD: *p*-value for the comparison of control vs doxorubicin; pCxL: *p*-value for the comparison of control vs. liraglutide; pDxDL: *p*-value for the comparison of doxorubicin vs doxorubicin + liraglutide; pLxDL: *p*-value for the comparison of liraglutide vs doxorubicin + liraglutide.

The Shannon index, an indicator of community richness, did not differ between the groups before and after the treatments (Figure 6). Before the treatment, β-diversity was similar between the groups (Figure S2) and after the treatment, it was different between groups C and D, C and L, L and DL. Also, there was a trend toward modulation of the gut microbiota in D group with the administration of liraglutide (Figure 7).

**Figure 8:**
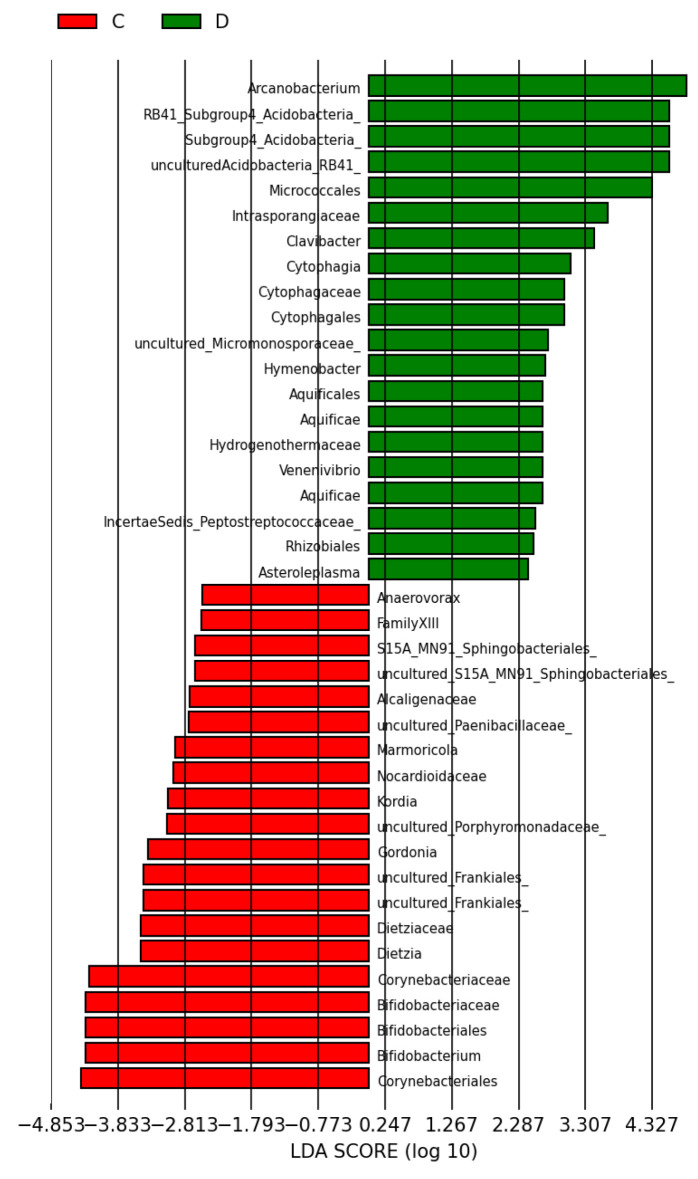
Linear discriminant analysis (LDA) score: overall structure and composition of the gut microbiota differences between control and doxorubicin groups after treatment. Positive and negative LDA scores indicate the dominant species in groups D and C, respectively. The histogram shows the lineages with LDA values > 2.0 and with *p* < 0.05.

**Figure 9:**
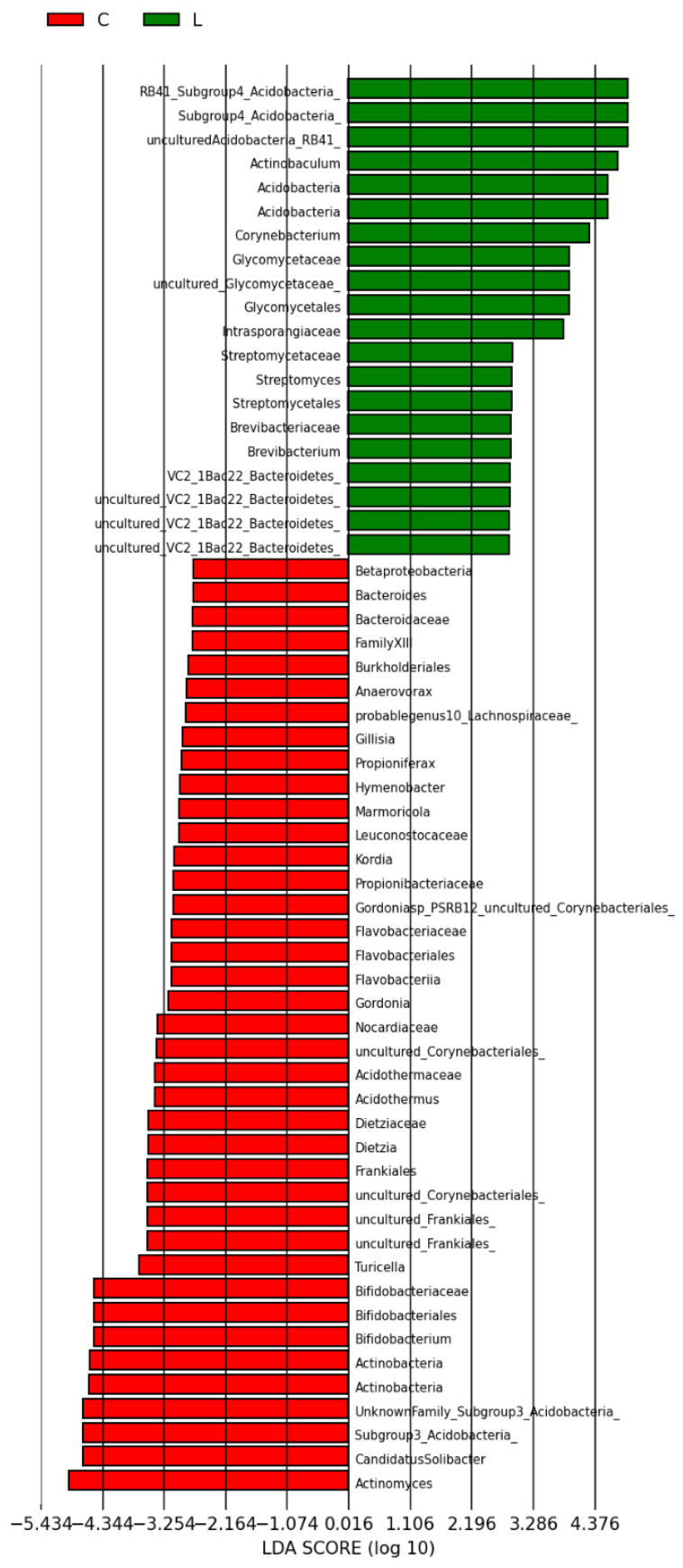
Linear discriminant analysis (LDA) score: overall structure and composition of the gut microbiota and differences between control and liraglutide groups after treatment. Positive and negative LDA scores indicate the dominant species in groups L and C, respectively. The histogram shows the lineages with LDA values > 2.0 and with *p* < 0.05.

**Figure 10:**
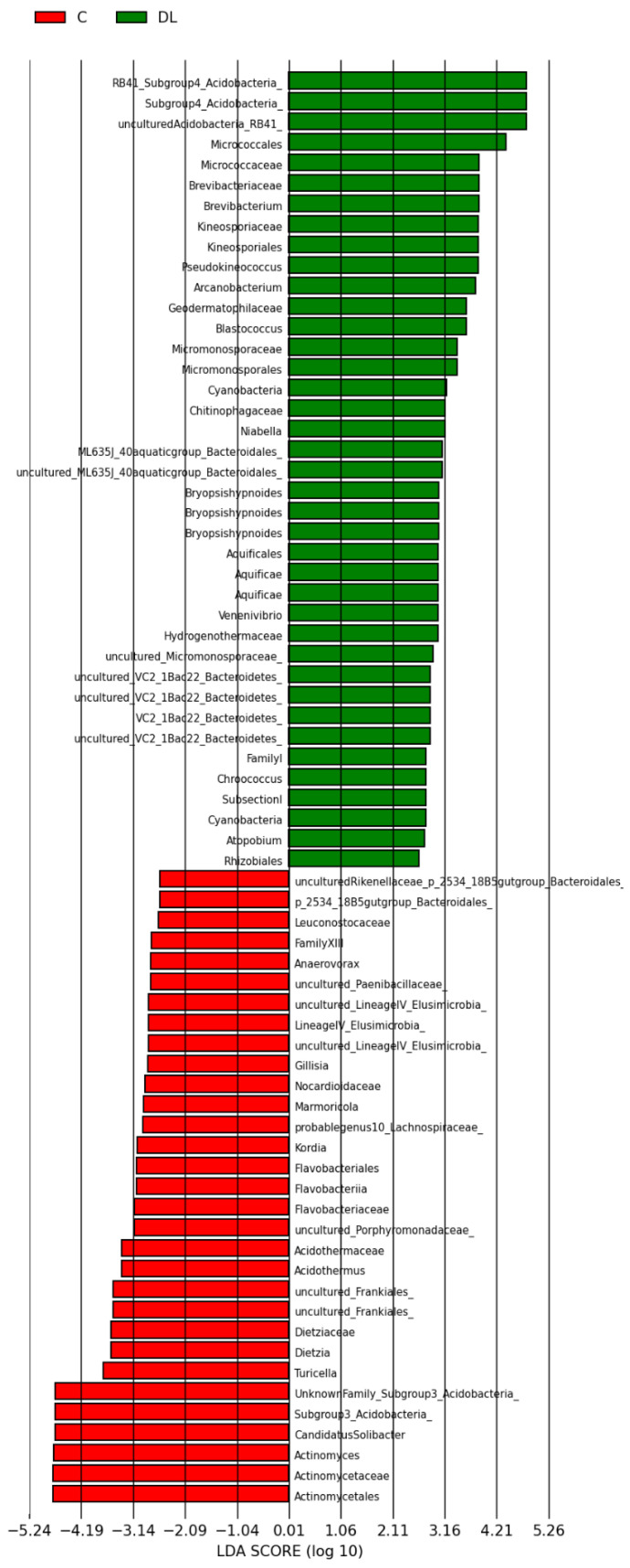
Linear discriminant analysis (LDA) score: overall structure and composition of the gut microbiota differences between control and doxorubicin + liraglutide groups after treatment. Positive and negative LDA scores indicate the dominant species in groups DL and C, respectively. The histogram shows the lineages with LDA values > 2.0 and with *p* < 0.05.

A total of 103 phylotypes were identified as high-dimensional biomarkers. The order Micrococales, the Intrasporangiaceae family, the Acidobacteria phylum, and the genera Arcanobacterium and Clavibacter were more prevalent in group D than C (Figure 8). In addition, the abundance of the Acidobacteria phylum and the genera Corynebacterium and Actinobaculum were higher in group L than C (Figure 9). Finally, the abundance of the order Micrococales, the families Brevibacteriaceae and Kineosporiaceae, the Acidobacteria phylum, and the genus Arcanobacterium was higher in group DL than L (Figure 10).

### 3.7. Fecal SCFAs

**Figure 11:**
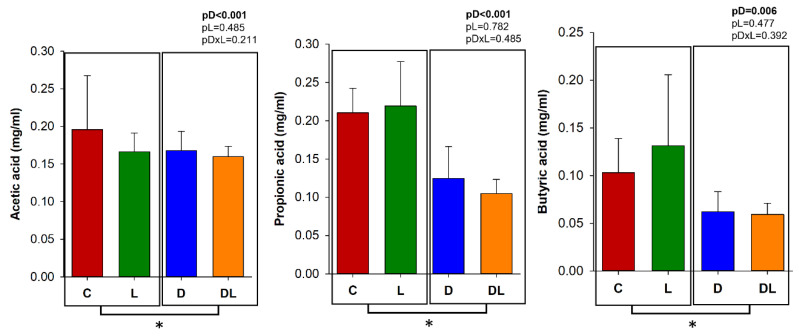
Measurement of fecal short-chain fatty acids (SCFAs) after treatment. C: control; D: doxorubicin; L: liraglutide; DL: doxorubicin + liraglutide. Sample size: 5 per group. Values are expressed as mean ± standard deviation. GLM; pDxL: *p*-value for the interaction between doxorubicin and liraglutide; pD: *p*-value for the doxorubicin factor; pL: *p*-value for the liraglutide factor; * different for the doxorubicin factor.

The concentrations of acetic, propionic, and butyric acid were measured in the feces before and after the treatments. Before treatment, fecal SCFA did not differ between the groups (Figure S3). After treatment, fecal concentrations of acetic, propionic, and butyric acids were lower in the rats treated with doxorubicin (D and DL) in comparison to those that did not receive doxorubicin (C and L; Figure 11).

## 4. Discussion

Doxorubicin is a widely utilized chemotherapeutic agent with proven efficacy against various malignancies. However, its use is frequently associated with both acute and chronic adverse effects, which can compromise patient compliance and ultimately reduce the overall effectiveness of treatment [38].

Pathophysiology of doxorubicin-induced toxicity is complex and involves multiple mechanisms, including inflammation, oxidative stress, mitochondrial dysfunction, apoptosis, intracellular calcium transient changes, and matrix metalloproteinase activation [39,40,41,42]. The most studied side effect is cardiotoxicity due to its association with the development of heart failure. The side effects on the gastrointestinal tract are less studied, but are also of great importance, possibly contributing to the maintenance and propagation of systemic inflammation [5].

In our study, we observed significant structural changes in the intestinal epithelium of doxorubicin-treated rats, as previously observed by other authors. Fan et al. observed partial loss of intestinal mucosal integrity, edema, glandular atrophy, and inflammatory infiltrate in rats with chronic doxorubicin toxicity [43]. Here, a single, acute doxorubicin dose of 75 mg/m^2^ increased intestinal permeability in pigs [44]. Zhen et al. observed increased levels of LPS and ZO-1 in patients treated with doxorubicin and degeneration of intestinal villi and crypts in mice treated chronically with the drug [45].

Doxorubicin-induced intestinal injury has been associated with inflammation [46], apoptosis [47], and oxidative stress [48]. However, in our work, the damage to the intestinal epithelia did not appear to have been predominantly mediated by apoptosis, inflammation, or oxidative stress, as the expression of Bcl-2, NFκB, TNF-α, TLR-4 and antioxidant enzymes remained unchanged. It is important to note that inflammatory responses are dynamic and may vary according to factors such as the dosing regimen, timing of tissue collection, and experimental conditions. Therefore, the absence of detectable changes in these markers at the evaluated time point does not necessarily exclude the occurrence of inflammatory processes but may reflect a different temporal profile or involvement of alternative pathways. Moreover, additional molecular mediators related to these mechanisms were not assessed in the present study.

As mentioned earlier, the gut microbiota has increasingly attracted the attention of researchers recently, as it appears to be associated with diseases such as inflammatory bowel disease, diabetes, atherosclerosis, obesity, and heart failure [14]. Studies have also evaluated the effects of different substances on doxorubicin-induced gut and cardiovascular toxicity [29,31,32,49,50]. As an example, donepezil administration increased ZO-1 and occludin levels and changed the gut microbiota β-diversity in doxorubicin-treated rats, suggesting an improvement in the integrity of the intestinal barrier [30].

In our study, α-diversity did not differ before and after treatment within each group. α-diversity is used to analyze variety and abundance of microbial species within specific groups, describing the richness, the total number of different species, and the evenness—in other words, how the species are distributed. Therefore, α-diversity represents the internal population diversity [51]. Our results are similar to the findings of studies in acute and chronic doxorubicin models, but other authors have shown that doxorubicin reduced α-diversity [31,32,43,52,53]. These differences in α-diversity may occur due to the diversity of the microbial community in different models, animals, and locations.

β-diversity refers to the difference in microbiota composition between groups, measuring the degree of dissimilarity [54]. Before treatment, β-diversity did not differ between groups, but at the end of the experiment, β-diversity was different between groups C, L, and D. Similar results have also been observed in other experimental studies with doxorubicin [32,52].

Interestingly, liraglutide alone altered the β-diversity, but no effect was seen when combined with doxorubicin. We did not find any studies that evaluated the effects of liraglutide or other GLP-1 analogs on the gut microbiota in relation to doxorubicin-induced toxicity. Researchers have shown that GLP-1 analogs modulate β-diversity in different conditions. For example, administration of liraglutide modulated β-diversity in diabetic patients [19], in high-fat-diet-fed mice [55], and in dehydroepiandrosterone-induced polycystic ovary syndrome mice [56]. Our study is the first to evaluate the effects of a GLP-1 analog on the gut microbiota in a model of doxorubicin toxicity.

Relative abundance refers to the proportion of specific bacteria in a bacterial community [57]. As expected, the predominance of the Firmicutes and Bacteroidetes phyla was initially observed in all groups. After treatment, the abundance of the Bacteroidetes phylum was lower in the rats in groups L and D compared to C. The Bacteroidetes phylum is constituted mainly of Gram-negative bacteria, which are important for digesting complex polymers, facilitating food digestion, and nutrient absorption [58]. They produce butyrate, a short-chain fatty acid that helps maintain the intestine barrier. Bacteroidetes also participate in T cell immunomodulation, interleukin-8 down-regulation, and a reduction in pathogenic bacteria colonization [59,60]. Thus, a decrease in this phylum may have contributed to the intestinal injury.

Our study is one of the first to show that doxorubicin reduces the abundance of the Bacteroidetes phylum in the gut microbiota. Previous studies have shown that depletion of Bacteroidetes was associated with inflammatory bowel disease [61], coronary artery disease [62], atherosclerosis [63] and heart failure [64]. The effect of liraglutide on the Bacteroidetes phylum is unclear, as liraglutide reduced its abundance while increasing the prevalence of Firmicutes in polycystic ovary syndrome mice [56]. On the other hand, liraglutide had no effect on Bacteroidetes, while reducing Proteobacteria and increasing Verucomicrobia in high-fat-diet-fed mice [65].

Additionally, the abundance of Proteobacteria was higher in the rats that received doxorubicin, as seen previously in rodents chronically treated with doxorubicin [31,32]. This phylum increases in gut inflammation models, particularly inflammatory bowel disease [66]. Proteobacteria are the greatest producers of LPS, molecules present in the outer cell membrane of Gram-negative bacteria [67], and responsible for initiating local and systemic inflammatory responses through the activation of toll-like receptors. Thus, considering the increase in Proteobacteria in animals treated with doxorubicin and the intestinal barrier damage observed in histology, we expected an increase in serum LPS, as previously reported [46]. However, we did not observe differences in LPS serum concentration in our study. It is possible that other microbial products, including nucleic acids, can activate toll-like receptors and contribute to intestinal inflammation and injury [46]. For example, novel markers have recently been introduced to evaluate intestinal barrier damage, such as the zonulins, proteins that participate in the tight junctions [68].

In our LDA score analyses, the treated groups had a higher abundance of the phylum Acidobacteria than the control. This phylum is composed mainly of Gram-negative bacteria. Although there is limited research on the role of Acidobacteria in organisms, they have been identified in inflammatory bowel disease patients [69]. In groups D and DL, the genus Arcanobacterium predominated, Gram-positive bacteria known for causing upper respiratory tract infections. However, we did not identify any studies that characterized this genus in the context of gastrointestinal disease [70]. In group D, the genus Clavibacter, aerobic Gram-positive bacteria, predominated, which has only ever been associated with changes in agricultural products without any pathogenic significance in humans to date. Finally, group L had a higher predominance of the genera Corynebacterium and Actinobaculum than group C; both are composed of Gram-positive bacteria. The first genus colonizes the skin and mucous membranes in humans, while the second is associated with urinary tract infections [71,72].

The animals treated with doxorubicin presented lower fecal concentration of butyric, acetic, and propionic acids than the rats that did not receive doxorubicin, as similarly seen by Wu et al. in mice with acute doxorubicin toxicity. SCFAs are metabolites of dietary products produced by the gut microbiota, especially bacteria from the phylum Firmicutes, followed by Actinobacteria, Bacteroidetes, Proteobacteria, and Verrucomicrobia [31]. SCFA play important roles in providing energy to enterocytes, attenuating production of pro-inflammatory mediators, and modulating innate immune cell’s function, mucin production, and expression of cell junction proteins [15]. Thus, they ensure the integrity of the intestinal barrier [9,73].

The reduction in fecal SCFA concentrations observed in doxorubicin-treated animals should be interpreted within a multifactorial physiological context. Doxorubicin administration was associated with marked reductions in food intake and body weight, which may have decreased the availability of fermentable substrates in the intestinal lumen and thereby contributed, at least in part, to lower SCFA production. Nevertheless, SCFAs are widely recognized as key functional indicators of gut microbial metabolic activity and intestinal homeostasis. Therefore, the observed decrease likely reflects not only indirect effects related to treatment-induced hypophagia, but also a broader disruption of intestinal metabolic balance and host–microbiota interactions associated with chemotherapy-induced toxicity.

Importantly, animals treated with liraglutide also exhibited reduced body weight at the end of the experimental period, yet fecal SCFA levels remained unchanged in this group. This finding suggests that body weight reduction alone may not fully account for the SCFA decline observed following doxorubicin exposure. Rather, the results support the interpretation that doxorubicin-induced intestinal injury and microbiota alterations may have contributed to impaired microbial metabolic output. In contrast, liraglutide administration did not significantly modify fecal SCFA concentrations under the experimental conditions evaluated. To date, evidence regarding the effects of GLP-1 receptor agonists on fecal SCFA levels remains limited, and further mechanistic studies are warranted to clarify the physiological significance of these observations.

This study has some limitations that should be acknowledged. First, classical functional markers of intestinal barrier integrity, such as direct assessment of intestinal permeability were not evaluated, which limits a more comprehensive understanding of the physiological mechanisms underlying the observed findings. Second, the experimental design focused on an acute model of doxorubicin-induced intestinal toxicity, and therefore the long-term effects of liraglutide on intestinal structure, microbiota dynamics, and systemic outcomes could not be determined. In addition, although alterations in gut microbiota composition and metabolic activity were identified, the present study was not designed to establish causal relationships between microbial changes and intestinal injury or recovery. Future investigations incorporating mechanistic approaches, longitudinal analyses, and functional validation strategies will be important to further elucidate the role of host–microbiota interactions in chemotherapy-induced toxicity and to better define the therapeutic potential of GLP-1 receptor agonists in this context.

## 5. Conclusions

Acute doxorubicin toxicity was associated with marked structural injury to the large intestine, disruption of gut microbiota composition, and reduced fecal short-chain fatty acid production, highlighting the multifactorial nature of chemotherapy-induced intestinal toxicity. Although liraglutide pretreatment was associated with modulation of gut microbiota profiles, it did not mitigate the histological intestinal damage observed in this acute experimental model. To the best of our knowledge, this is the first study to investigate the potential influence of a GLP-1 receptor agonist on gut microbiota and intestinal toxicity in the context of doxorubicin administration.

## Data Availability

The data for this study have been deposited in the European Nucleotide Archive (ENA) at EMBL-EBI under the accession number PRJEB90078 (https://www.ebi.ac.uk/ena/browser/view/PRJEB90078) (accessed on 5 June 2025).

## Supplementary Materials

The following supporting information can be downloaded at: https://www.mdpi.com/article/10.3390/antiox15050538/s1, Figure S1: Relative abundance in the gut microbiota at the phyla level before treatment. Figure S2: β-diversity before treatment. Principal Coordinate Analysis (PCoA). Figure S3: Measurement of fecal short-chain fatty acids (SCFAs) before treatment.
