# Supplementary material for: Liraglutide Modifies Gut Microbiota Without Modulating Doxorubicin-Induced Toxicity in Rats
Source: Antioxidants (Basel). 2026 Apr 24;15(5):538. doi: 10.3390/antiox15050538 (PMC13203684; doi:10.3390/antiox15050538)
Supplement: Supplementary file 1 [file antioxidants-15-00538-s001.zip › antioxidants-4133470-supplementary.pdf]

SUPPLEMENTARY MATERIAL

Prior to any treatment, the two most abundant phyla were *Firmicutes* (C 75.5 ± 23.4; D 80.8 ± 45.4; L 84.3 ± 16; DL 86 ± 12.7%; pD = 0.60; pL = 0.29; pDxL = 0.79) and *Bacteroidetes* (C 19.1 ± 18.5; D 17 ± 1.82; L 8.9 ± 10.3; DL 9.67 ± 8.84 %; pD = 0.90; pL = 0.11; pDxL = 0.79), with no statistically significant differences between the groups.

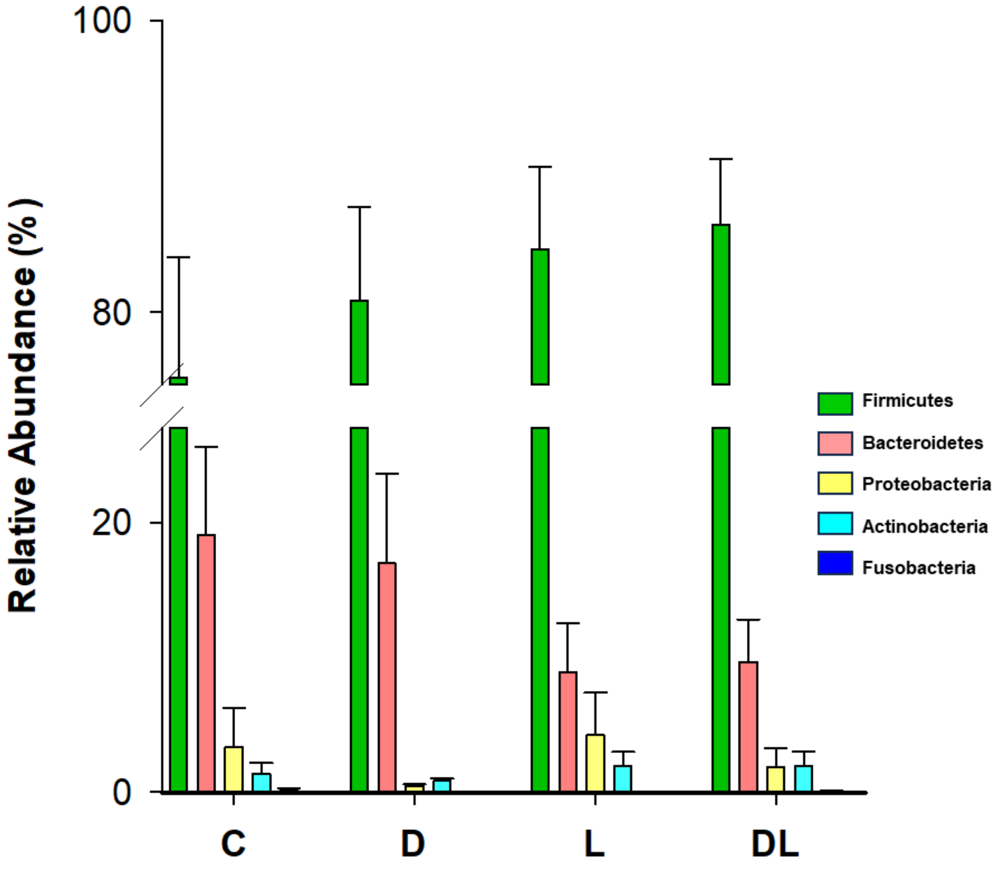

Figure S1. Relative abundance in the gut microbiota at the phyla level before treatment. C: control; D: doxorubicin; L: liraglutide; DL: doxorubicin + liraglutide. Data are expressed as means ± standard deviation.

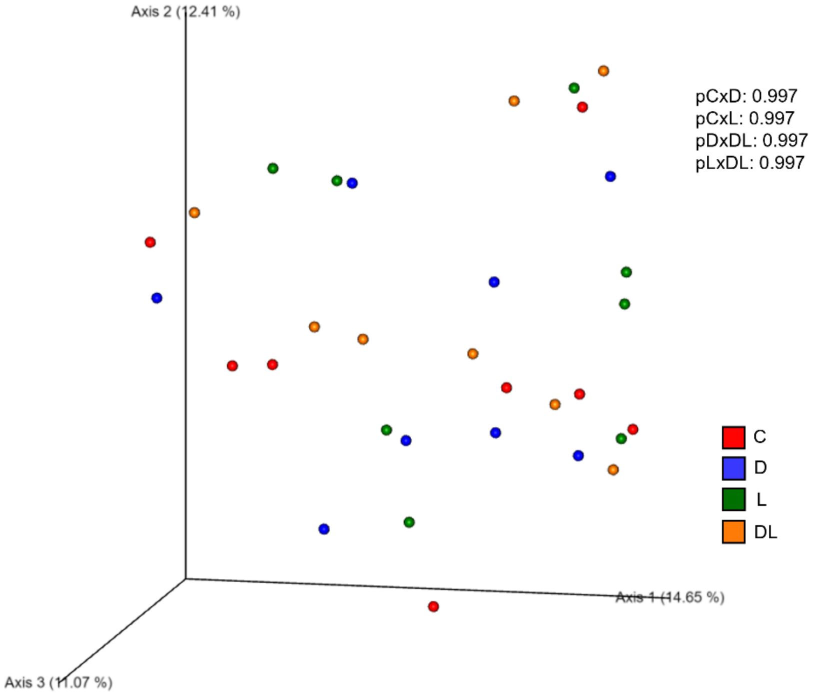

Figure S2. β-diversity before treatment. Principal Coordinate Analysis (PCoA). C: control; D: doxorubicin; L: liraglutide; DL: doxorubicin + liraglutide. Sample size: 8 per group. PERMANOVA; pCxD: p-value for the comparison of control vs. doxorubicin; pCxL: p-value for the comparison of control vs liraglutide; pDxDL: p-value for the comparison of doxorubicin vs. doxorubicin + liraglutide; pLxDL: p-value for the comparison of liraglutide vs doxorubicin + liraglutide.

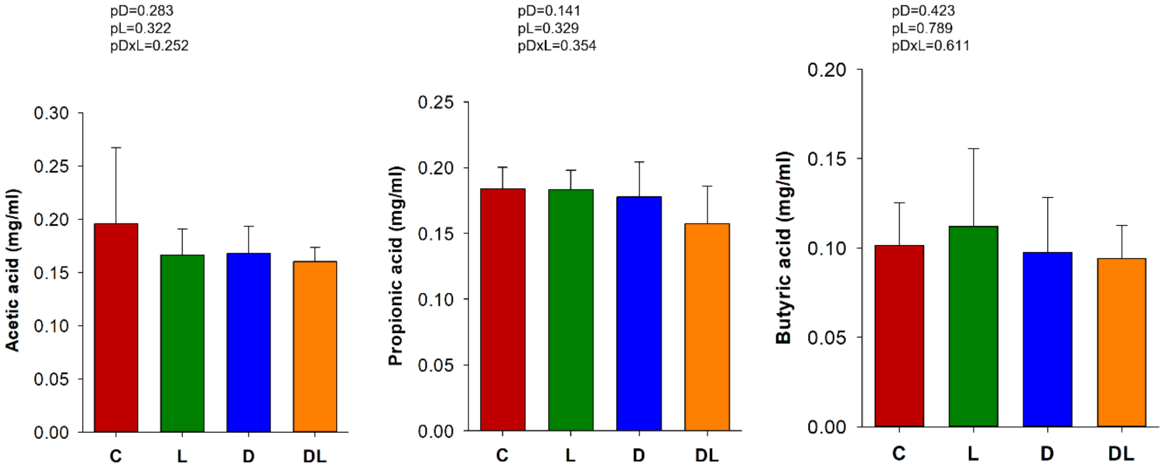

Figure S3. Measurement of fecal short-chain fatty acids (SCFAs) before treatment. C: control; D: doxorubicin; L: liraglutide; DL: doxorubicin + liraglutide. Sample size: 5 per group. Values are expressed as mean ± standard deviation. GLM; pDxL: p-value for the interaction between doxorubicin and liraglutide; pD: p-value for the doxorubicin factor; pL: p-value for the liraglutide factor.

Antibody information

Primary Antibody	Type	Reference	Dilution
TNF- α	Mouse monoclonal IgG	SC-52746	1:500
NFκB	Mouse monoclonal IgG	SC-8008	1:200
p-NFκB	Mouse monoclonal IgG	SC-136548	1:200
TLR-4	Mouse monoclonal IgG	SC-293072	1:200
BCL-2	Mouse monoclonal IgG	SC-7382	1:200
Anti-mouse	Rabbit anti-mouse IgG	AB 6728	1:3000 to 1:2000
